# Atomic-scale decoration for improving the pitting corrosion resistance of austenitic stainless steels

**DOI:** 10.1038/srep03604

**Published:** 2014-01-08

**Authors:** Y. T. Zhou, B. Zhang, S. J. Zheng, J. Wang, X. Y. San, X. L. Ma

**Affiliations:** 1 Shenyang National Laboratory for Materials Science, Institute of Metal Research, Chinese Academy of Sciences, Wenhua Road 72, 110016 Shenyang, China; 2 These authors contributed equally to this work.; 3 Current address: Materials Physics and Applications Division, MPA-CINT, Los Alamos National Laboratory, Los Alamos, NM 87545, USA.

## Abstract

Stainless steels are susceptible to the localized pitting corrosion that leads to a huge loss to our society. Studies in the past decades confirmed that the pitting events generally originate from the local dissolution in MnS inclusions which are more or less ubiquitous in stainless steels. Although a recent study indicated that endogenous MnCr_2_O_4_ nano-octahedra within the MnS medium give rise to local nano-galvanic cells which are responsible for the preferential dissolution of MnS, effective solutions of restraining the cells from viewpoint of electrochemistry are being tantalizingly searched. Here we report such a galvanic corrosion can be greatly resisted via bathing the steels in Cu^2+^-containing solutions. This chemical bath generates Cu_2−δ_S layers on the surfaces of MnS inclusions, invalidating the nano-galvanic cells. Our study provides a low-cost approach via an atomic scale decoration to improve the pitting corrosion resistance of stainless steels in a volume-treated manner.

Stainless steels, widely used in industry and in our daily life, are in fact not “stainless”. In the presence of aggressive anionic species, they are susceptible to the localized pitting corrosion that is one of the major causes of the materials' failure, leading to a huge loss to our society. Therefore, to clarify the origin of pitting process and to improve the pitting resistance in stainless steels has been one of the major research focuses in several disciplines such as materials science, chemistry, and metallurgy^1^,^2^,^3^,^4^,^5^.

Sulphur, particularly in austenitic stainless steels, plays critical roles in tuning the material's properties. For example, manganese sulphide (MnS) as a major existence of sulphur in stainless steels taking a role of lubricant is needed for machining and extending cutting tool life^6^. However, high sulphur content, and thus high volume of MnS in stainless steels, results in poor pitting resistance, since it is generally believed that the pitting events originate from the local dissolution of MnS^7^,^8^,^9^,^10^,^11^. Our recent studies^12^,^13^ based on high-resolution transmission electron microscopy (HRTEM) indicated that the MnS inclusion is not only compositionally inhomogeneous but also structurally dissimilar. We found that novel MnCr_2_O_4_ nano-octahedra embedded in the MnS medium catalyzed the local dissolution of MnS by the local nano-galvanic cells of MnCr_2_O_4_/MnS. Since then, effective solutions of restraining the cells from electrochemically working have been being tantalizingly searched.

However, invalidating the cells in a low-cost and high-efficiency way has been unattainable with current treatments performed both in industry and laboratories. Usually, final annealing and the subsequent pickling performed on the finished products are the two most common processes to enhance the localized corrosion resistance of stainless steels. During the former processes, the improvement of corrosion resistance is ascribed to the oxidation of steel surface^14^. While the principle beneficial factor of the latter process is the removal of MnS inclusions^15^. Nevertheless, the crevices and residual sulphide after removal of MnS were still identified as the sensitive sites for pitting initiation^16^. Other particular approaches, such as eliminating the MnS at the steel surface by laser or high current pulsed electron beam surface treatment^10^,^17^,^18^ which have been tried in laboratories and to some extend enhanced the pitting resistance, are of high-cost and low-efficiency. Their applications are believed to be rather limited in volume-treated industrialized processes.

It is known that the composition of sulphide inclusions plays a critical role in the initiation of pitting corrosion of stainless steels^19^,^20^. For instance, Cu-containing MnS inclusions are found to be electrochemically inactive in initiating pitting corrosion^8^. Besides, alloying Cu could eliminate the harmful sulphur species in corrosive solution by producing insoluble Cu_2_S^21^,^22^. In the present study, we synthesized, in virtue of the cation exchange reaction^23^,^24^,^25^,^26^, a thin film of Cu_2−δ_S on the exposure layer of MnS. Such chemical decoration leads to the inactivation of the anodic MnS inclusions and avoiding the nano-galvanic cells from electrochemically working, by which the pitting resistance of austenite stainless steels is significantly enhanced.

## Results

### MnCr_2_O_4_ nano-octahedron catalyzing local dissolution of MnS in stainless steels

**Figure 1:**
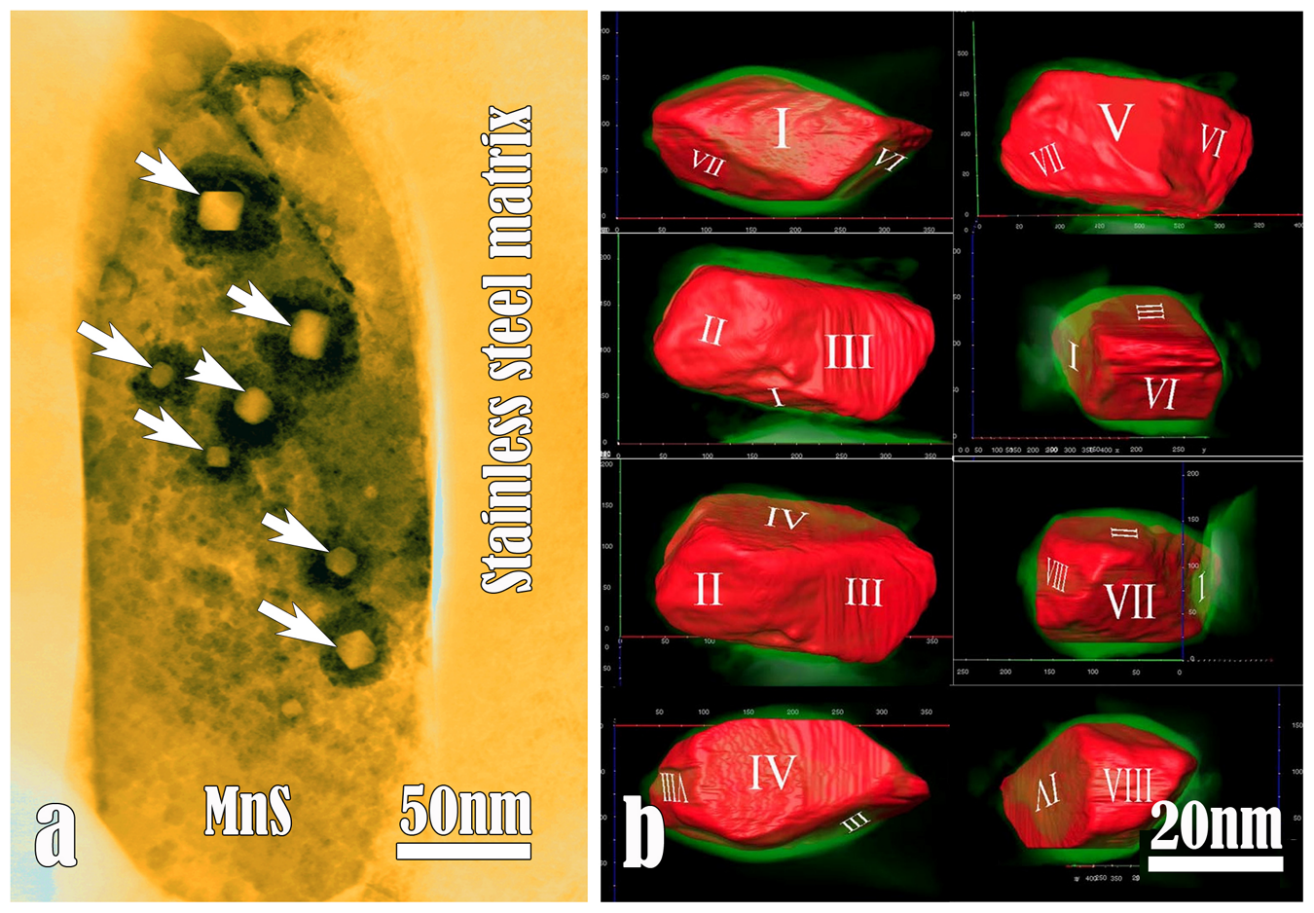
Identification of nano-octahedra as the initial sites where MnS starts to be dissolved. (a) A HAADF image showing the MnCr_2_O_4_ nano-particles (arrowed) catalyzed the local dissolution of a MnS inclusion section. Extensive studies based on various TEM techniques indicated that a single MnS inclusion is compositionally inhomogeneous and structurally dissimilar. Usually in each MnS inclusion, a number of MnCr_2_O_4_ nano-particles are embedded, and each oxide particle acts as a “tumour” making MnS locally be dissolved. (b) Determination of octahedron by 3D tomography technique. Each of the oxide particle has an octahedron configuration enclosed by eight {111} planes which are labeled with I, II, III, … VIII, respectively.

A commercial hot-rolled 316F stainless steel with high sulphur content was chosen since it provides a large number of MnS inclusions for analysis. The mechanical rolling made MnS inclusions needle-shaped and parallel to the rolling direction. Dimensions of the MnS inclusions are several tens of micrometers in length and a few hundred nanometers in diameter. MnS has a NaCl-type structure with lattice parameter of 0.52 nm. TEM observations were performed under the newly developed high-angle-angular-dark-field (HAADF) technique, which provides strong contrast associated with the local variety of chemical composition (atomic number) and/or thickness contribution by detecting high-angle scattering electrons^27^. As reported in the earlier study^12^, the dissolution of MnS is strongly localized in salt water: the pit which results from the local dissolution of MnS always features a nano-scale undissolved core whose structure and composition are different from that of MnS. Namely, the initial site of MnS dissolution is at the periphery of a nano-particle which was embedded in MnS medium (Fig. 1a).

Based on a series of EDPs and energy dispersive X-ray spectroscopy (EDS), the nanoparticles are identified to be spinel MnCr_2_O_4_, or Mn(Cr,Ti)_2_O_4_ if small amount of titanium is taken into account. By means of large-angle tilting experiments and 3D tomography in the TEM, we found that each of the MnCr_2_O_4_ nano-particles has a specific geometric shape, which was identified basically to be an octahedron enclosed by eight triangles. The 3D morphology of an octahedron is displayed in Fig. 1b where each of the {111} planes is alternately exhibited. It is worthwhile to mention that not all the MnCr_2_O_4_ nano-particles feature a regular octahedron-shaped, as that shown in our previous study. In some cases, they are octahedron but with remarkable distortion, shown in Fig. 1b. This indicates that the detailed atomic configurations at the octahedron surface are quite complicated.

The spinel MnCr_2_O_4_ particles shown here, although they are as fine as nano-meter scale, are present in almost all the MnS inclusions. So far, little attention has been paid to the ultra-fine inclusions in stainless steels, which is based on the cognition that they do not undermine the mechanical properties of the steels. However, the nano-sized oxide particles shown above are found to play a critical *chemical* role in catalyzing MnS dissolution and pitting corrosion of the steels.

### Chemical modification improving the pitting resistance

**Figure 2:**
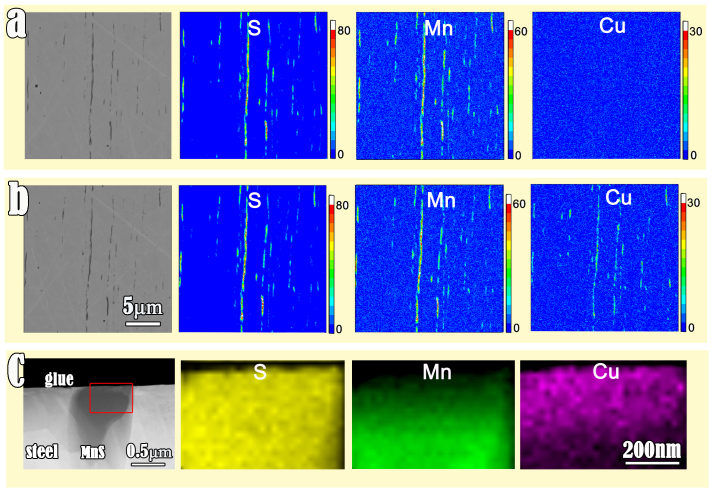
The elemental distribution on the surfaces of steel samples. (a) and (b) SEM and EPMA images of the same steel sample surface before (a) and after (b) chemical modification in 0.01 M CuSO_4_ solution for one hour, respectively. According to the SEM images of the sample (a) before and (b) after chemical modification in 0.01 M CuSO_4_ solution for one hour, little difference can be identified in between. However, remarkable change of MnS inclusions can be clearly noted by comparisons of elemental maps. Except Mn and S, primary MnS has no signal of Cu. In contrast, after chemical modification, besides Mn and S, the Cu signal is visible in the “MnS inclusions”. (c) cross-sectional HADDF image and EDS elemental mapping obtained in the sample treated in 0.05 M CuSO_4_ solution for 1 h. The copper sulphide layer was about 200 nm thick. This composition identification indicates that cation exchange reaction occurred during chemical bath of the steel sample.

The chemical modification of MnS inclusions was performed by immersing the polished stainless steel sheets in a dilute CuSO_4_ solution at room temperature. The electron-probe X-ray microanalysis (EPMA) experiment clearly reveals that the cation exchange reaction has taken place during the immersion, namely, Cu ions have selectively substituted some Mn^2+^ in MnS inclusions after 1 hour immersion (Fig. 2). Since the depth of characteristic X-rays produced by electrons is about one or two micrometers for the elements with medium atomic mass^28^, the whole MnS with diameter of several hundred nanometers is in the detection range of EPMA. Therefore, the co-existence of Mn and Cu signals in Fig. 2b indicates that the elements replacement should happened on the skin layer of MnS. Thanks to the passive film of stainless steels, iron has no obvious replacement reaction with copper ions since we did not find any morphology or composition change on the steel matrix. The elements substitution and the thickness of reaction layer are determined by the TEM cross-sectional observation of the treated sample which provides direct information on morphology and chemical compositions after the reaction. After the bulk sample was bathed in 0.05 mol/L CuSO_4_ solution for 1 h, a HAADF image and EDS elemental mapping of the selected area are shown in Fig. 2c. It is seen that the surface layer of MnS almost all transformed to copper sulphide. The thickness of copper sulphide layer was nearly 200 nm. A sharp interface cannot be distinguished between manganese sulphide and copper sulphide since the reaction process was controlled by cation diffusion.

**Figure 3:**
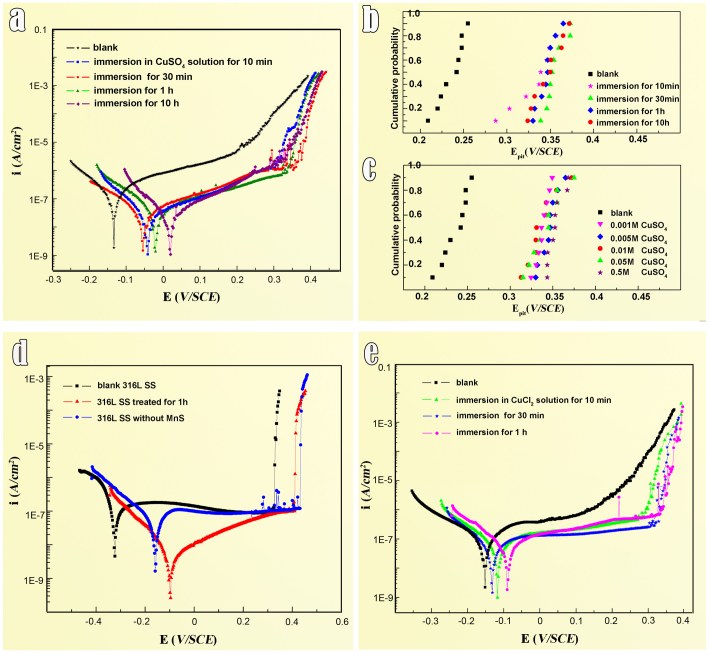
The potentiodynamic polarization tests and pitting potential distributions of the samples before and after chemical modification. (a) Polarization curves of 316F SS samples before and after treatment for various periods in 0.005 M CuSO_4_ solution. (b) The distribution of pitting potential of 316F SS before and after treatment for various periods in 0.005 M CuSO4 solutions. It seems that the pitting potentials remain no much changes during the bathing duration from 0.5 to 10 hours. This indicates that bathing as short as 30 minutes in above solutions are effective enough to greatly improve the corrosion resistance of the present stainless steels. (c) The distribution of *E_pit_* values of the samples treated in CuSO_4_ solution with various concentrations for 1 hour. (d) Polarisation curves of 316L SS sample with MnS inclusion on surface before (black) and after treatment for one hour in 0.005 M CuSO_4_ solution (red). Corrosion behavior of the MnS-free 316L sample (blue) was also tested as comparison. Via chemical modification, the pitting potential of the treated 316L sample with MnS inclusions shifted to the same level as that of the MnS-free sample. (e) Polarisation curves of 316F SS sample before and after the treatment for various periods in 0.003 M CuCl_2_ solution. The chemical modification in CuCl_2_ solution has the same beneficial effect on enhancing the pitting resistance of stainless steel as that in CuSO_4_ solution.

To evaluate the enhancement of pitting resistance after chemical treatment, the potentiodynamic polarization tests on untreated and treated samples were performed in 0.5 mol/L NaCl electrolyte. Some typical potentiodynamic polarization curves are shown in Fig. 3a. It can be seen that all the treated samples feature a noble shift of the open circuit potentials (*E_ocp_*) and pitting potential (*E_pit_*) and an obvious decrease of passive current density, which indicates the chemical treatment results in an improved pitting resistance. Generally, the pitting potential data of stainless steel is scattered. To evaluate the beneficial effect of the chemical treatment in a statistic analysis, we tested many samples, including untreated and treated samples under different treating conditions. The distributions of *E_pit_* values for the samples before and after different treatment were plotted as illuminated in Figs. 3b and 3c. Cumulative probability shown in the vertical was calculated by a mean rank method: *P_cum_* = *i*/(*N* + *1*), where *P_cum_* is the cumulative probability of measured pitting potential (*E_pit_*), *i* is the order in the total number *N* (*i* = 1, 2, 3, …, *N*). In Fig. 3b, it is found that the onset of stable pitting on the untreated samples occurs at near 230 mV, while for the samples treated in CuSO_4_ solution with duration from 10 min to 10 h, all *E_pit_* shift to noble values by 100 mV on average. The treating duration longer than half an hour seems not to have more positive effect. It is probably because the cation exchange reaction reaches the equilibrium state in 30 minutes. We have carried out systemic electrochemical tests on the samples experienced bathing in CuSO_4_ solution with variant concentrations, like 0.001 mol/L, 0.005 mol/L, 0.01 mol/L, 0.05 mol/L, and 0.5 mol/L. The pitting potential of the samples treated in solution with different concentrations is shown in Fig. 3c. It is seen that the E_pit_ values of the samples, treated in such a wide range of solution concentrations, display similar.

For comparison with other steels, the pitting potential of a commercial 316L stainless steel sample was determined which is about 320 mV (as shown in Fig. 3d). Obviously, the chemical treatment makes 316F stainless steel win an equivalent level of pitting resistance with that of 316L stainless steel, which is generally believed to have an excellent corrosion resistance.

To confirm the universality of the chemical modification, we treated the 316L stainless steel in the Cu-containing solution and also found the treatment improved the pitting resistance as that in the 316F stainless steel; for instance, the *E_pit_* has a shift of nearly 100 mV after one hour treatment. As a comparison, small pieces of 316L sample with and without visible MnS inclusions, respectively, were selected for electrochemically testing. As seen from the potentiodynamic polarization curves (Fig. 3d), the chemically modified 316L sample with MnS inclusions is very close to that of its MnS-free counterpart, which indicates that such a chemical modification make MnS inclusions inactive although the spinel nano-oxides are still there.

In addition to the CuSO_4_ solution, dilute CuCl_2_ solutions with various concentrations are also applied for the chemical modification of MnS, and almost the same enhancement is achieved as that in CuSO_4_ (Fig. 3e).

### Chemical modification substantially lowering the pitting nucleation rate

**Figure 4:**
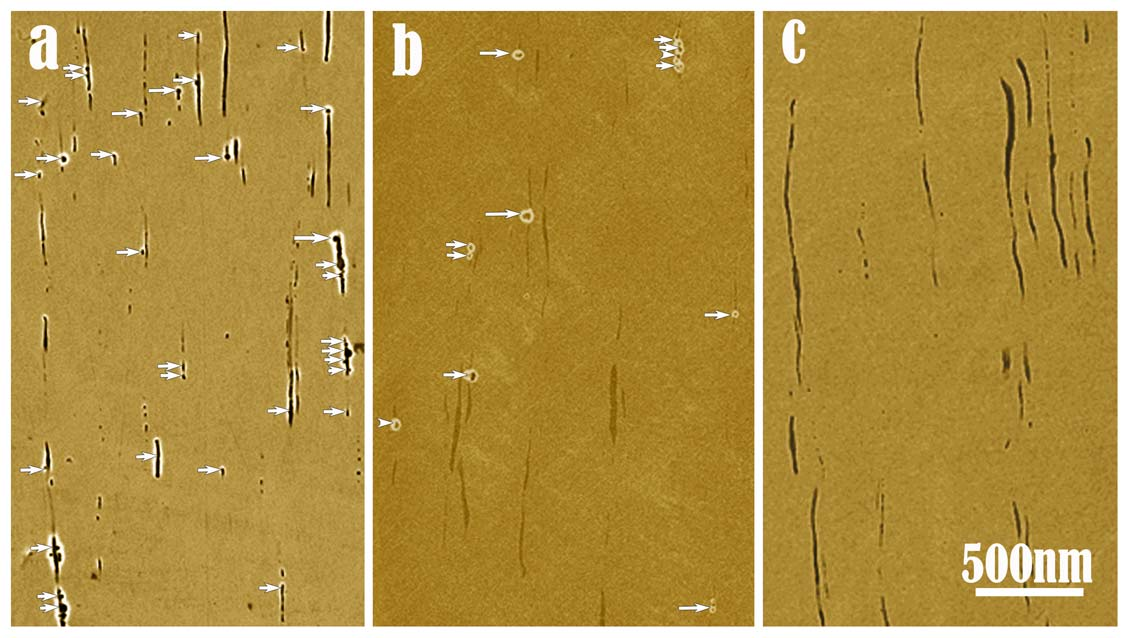
Typical SEM images of the samples suffered corrosion electrochemical tests in 0.5 M NaCl solution: (a) untreated sample, (b) sample with 10 minutes treatment in 0.005 M CuSO_4_ solution and (c) sample with 1 hour treatment in 0.005 M CuSO_4_ solution. The chemical modification via bathing the sample in CuSO_4_ solution greatly reduced the number of pits resulting from local dissolution of MnS.

The samples undergoing different treatments were potentiodynamically polarized to the potential above *E_pit_*, and the tests were terminated when the anodic current density reached the value of 1 mA/cm^2^. Then all the samples were unloaded and examined by scanning electron microscopy (SEM). Figure 4 shows three typical SEM images of the samples suffered electrochemical tests. In the untreated sample, each MnS dissolves severely and a large number of pits appear in the vicinity (arrowed, in Fig. 4a). In contrast, in the sample treated in the Cu-containing solution for 10 minutes, the number of the pits decreases dramatically (arrowed, Fig. 4b). One-hour-treatment results in a further decrease of the number of pits (Fig. 4c). Moreover, no obvious dissolution of the MnS inclusions was observed on the surfaces of treated samples even after the stable pits appeared, which indicates that the nano-galvanic cells of MnCr_2_O_4_/MnS have been invalided by the chemical decoration.

**Figure 5:**
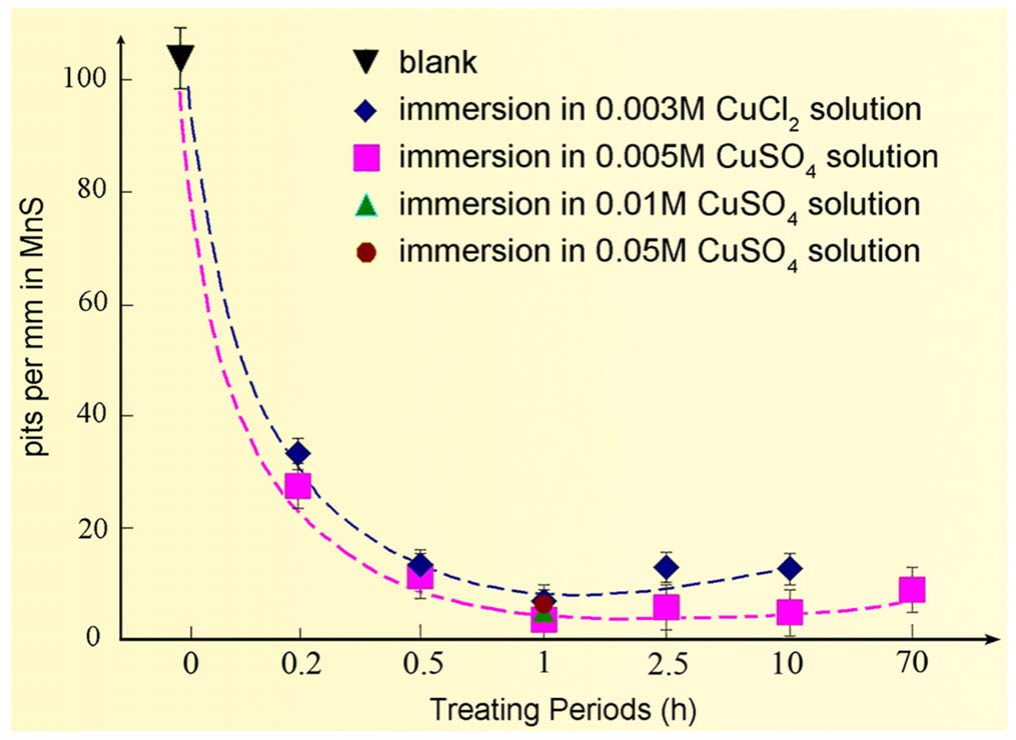
Statistical distributions of pits per unit length of MnS inclusion obtained from >300 inclusions on each sample surface. CuSO_4_ and CuCl_2_ solutions with various concentrations were used to bath the steel samples for variant durations. Whether CuSO_4_ or CuCl_2_ solution was applied, the numbers of the pits which results from the local dissolution of MnS are sharply reduced.

In order to characterize the substantial decrease of the number of pits on the chemically modified samples, the number of pits per unit length of the inclusion was statistically counted, based on over 300 MnS inclusions in each sample (Fig. 5). The minimum number reaches 4, approximately, on the sample experienced one hour immersion.

## Discussion

The general phenomenon of MnS dissolution is that it happens at the interface of MnS/MnCr_2_O_4_. The novel MnCr_2_O_4_ nano-octahedra embedded in the MnS medium catalyzed the local dissolution of MnS by the local nano-galvanic cells between them^12^,^13^. Once the dissolution process starts, the whole process goes rapidly. During the chemical modification in Cu^2+^-containing solution, the surface layer of MnS inclusions transforms into copper sulphide crystals, which is thought to be electrochemically inactive to initiate pitting corrosion^21^.

**Figure 6:**
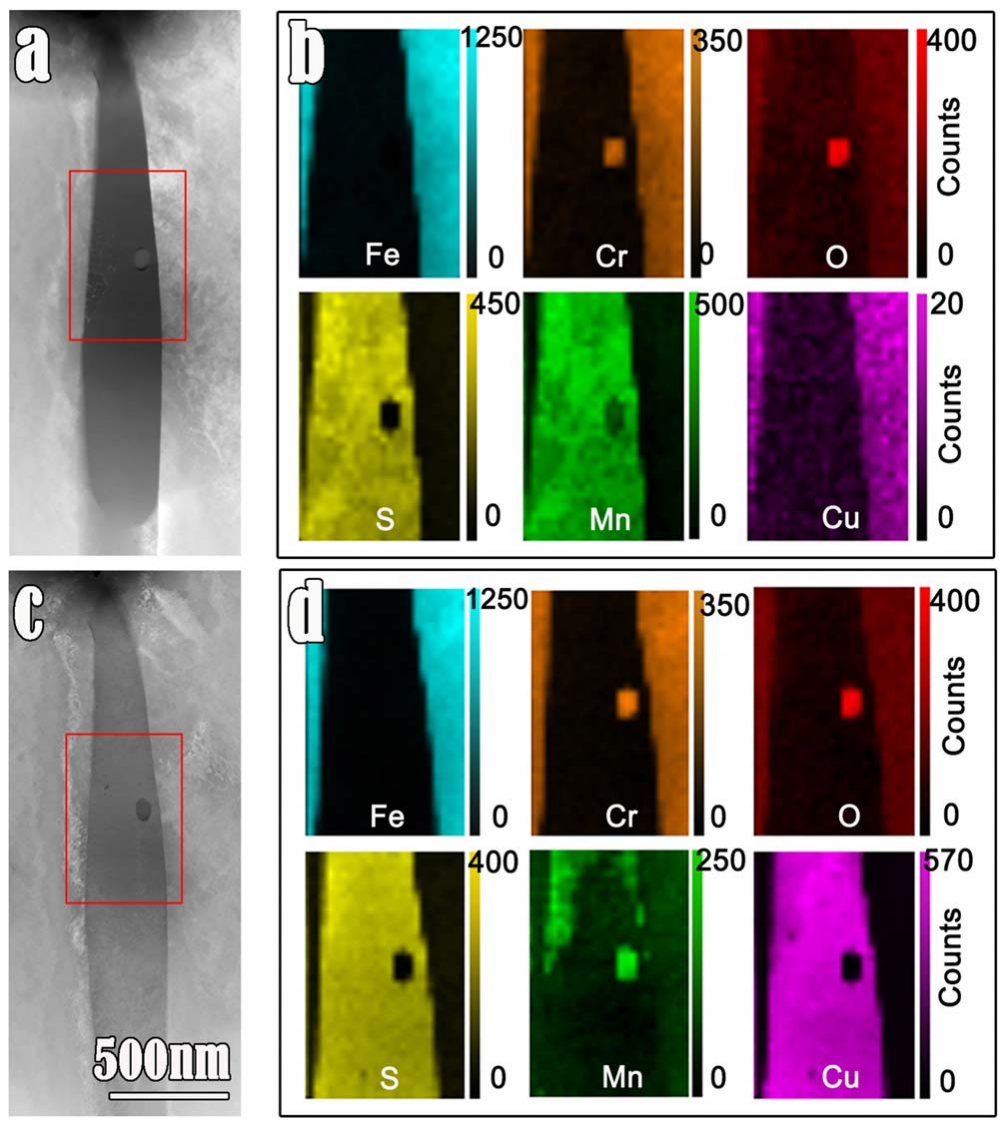
Comparison of the chemical composition before and after treatment. (a) HAADF image showing the morphology of steel matrix, MnS inclusion, and a MnCr_2_O_4_ particle within MnS. (b) EDS elemental maps of the area marked with red tangle in (a). The counts level on the right side of each map was drawn from EDS profiles and used to semi-quantify the elements content. (c) HAADF image of the same section as that in (a) but suffered the treatment in 0.05 M CuSO_4_ solution for 20 min. (d) EDS elemental maps of the area marked with red rectangle in (c). The MnS inclusion was almost transformed to copper sulphide.

**Figure 7:**
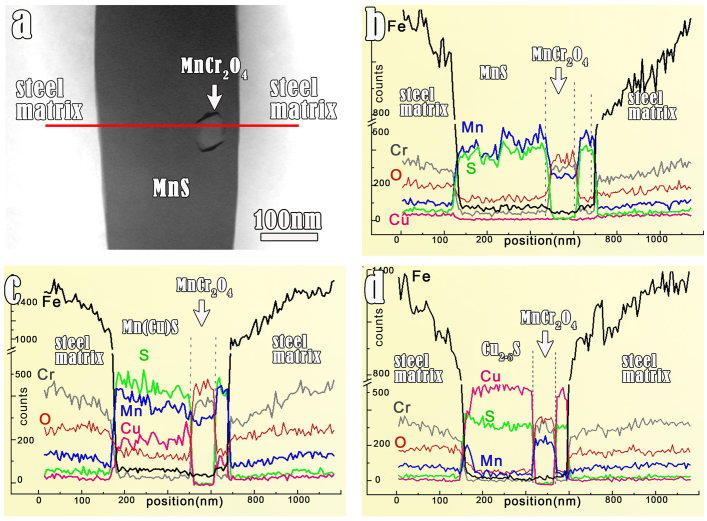
Analysis of composition evolvement in MnS versus variant durations of showering in the 0.05 M CuSO_4_ solution. A section of the sample is particularly focused. (a) HAADF image showing a MnS inclusion section with a MnCr_2_O_4_ particle therein. The horizontal red line marked in the image indicates the route along which chemical compositions are profiled. (b) The EDS profile of a scan made along the red line in (a). This profile corresponds to the chemical composition in the untreated sample. (c) EDS results along the same route but the sample experienced showering in CuSO_4_ solution for 10 minutes. Note that Cu signals get to appear in the “MnS inclusion”. (d) EDS results along the same route but the sample experienced showering in CuSO_4_ solution for 20 minutes. The intensity of Mn and Cu signals are reversed compared with that in (c) implying that most of Mn are replaced by Cu.

To figure out the details of reaction products, the TEM specimens before and after immersion in a CuSO_4_ solution for 20 minutes were observed, respectively. Detailed analyses of chemical compositions onto a section of a MnS inclusion (marked in Fig. 6a) were performed, as shown in Fig. 6b. It is seen that, initially, Cu is not present in the MnS inclusion. However, the composition of the same MnS inclusion (shown in Fig. 6c) exhibits a remarkable change after the chemical modification (Fig. 6d). Most of the MnS is replaced by copper sulphide, especially in the area where specimen thickness is only several tens of nanometers. The detailed chemical compositions analysis by means of EDS line scanning was performed. Fig. 7 displays one example of EDS analysis, where a MnCr_2_O_4_ nano-particle is embedded in MnS medium in the HAADF image (Fig. 7a). The EDS profiles (Fig. 7b–7d) are labeled which are obtained by line-scanning along the red line in Fig. 7a. The intensity of Cu-signal inside the inclusion gradually increases which suggests that the exchanged layer becomes thicker, with extending the immersion duration from 0, to 10 minutes and 20 minutes. Both steel matrix and the MnCr_2_O_4_ particle inside the MnS inclusion were noticed to remain unchanged during the process of chemical modification.

**Figure 8:**
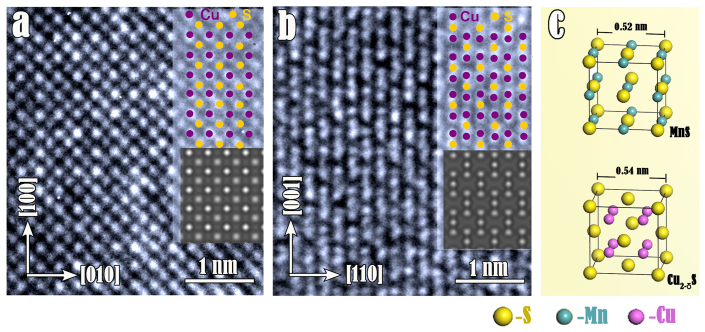
Identification of the product after the cation exchange reaction by aberration-corrected high-resolution transmission electron microscope. (a) HRTEM image along [001] direction of Cu_2−δ_S. The projection of the structure along [001] is superimposed on the image. The simulated image of the structure is shown as an inset with a thickness of 5.4 nm. Note that the copper columns in the image show brighter contrast in the image. (b) HRTEM image taken along 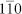 direction of Cu_2−δ_S. Atomic projection and simulated images are shown as insets. (c) The crystal cells of cubic MnS and Cu_2−δ_S. These two compounds share the same sub-lattice of S^2−^ anions and the lattice constant of them are rather close.

Determined by HRTEM images along [001] and 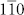 directions (Fig. 8a and 8b, respectively), the final products has an isotypic fluorite structure with lattice constant *a* = 0.54 nm. A combination of lattice parameter and chemical compositions concludes that such a compound corresponds to Cu_2−δ_S,namely digenite, of a stoichiometry approaching to Cu_1.8_S^29^. In such crystals, cations occupy the tetrahedral 8(c) sites and trigonal 32(f) sites randomly at room temperature. The crystal cells of cubic MnS and Cu_2−δ_S are displayed in Fig. 8c.

The final product Cu_2−δ_S indicates that both Cu^+^ and Cu^2+^ are involved in the cation exchange process. The Cu^+^ might be derived from the redox reaction between Cu^2+^ in CuSO_4_ eletrolyte and steel matrix. Although corrosion of stainless steels in dilute CuSO_4_ solution develops rather slowly^30^, it provides enough cuprous ions for the occurrence of cation exchange reaction at the free surfaces of MnS inclusions, since the area of the MnS inclusions is rather smaller compared with that of the whole steel surface. We propose the reaction pathway for the formation of Cu^+^ be written as follows: 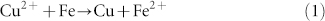

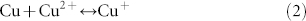


Since cubic Cu_2−δ_S and MnS share the same sub-lattice of S^2−^ anions and lattice constant of these two compounds are quite close, this enables the exchange between Cu^+^ and Mn^2+^ through the frame of S^2−^ anions without rearrangement of S^2−^ lattices (see Fig. 8c). In this reaction, the large difference of solubility (solubility product *K*_MnS_ = 10^−13^, *K*_Cu2S_ = 10^−48^) provides the thermodynamic driving force for the spontaneous ions replacement^26^. The ideal reaction is demonstrated as follows: 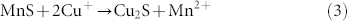
In the present study, the increase in pitting potential was attributed to the newly formed Cu_2−δ_S thin film which prevents the MnS from dissolution. In the galvanic system of MnS/MnCr_2_O_4_, the potential difference between MnS dissolution (*E*_MnS_) and oxygen reduction reaction (*E*_ORR_) provides the thermodynamic driving force. Anode MnS tends to dissolve, while ORR takes place on the cathode MnCr_2_O_4_. Once the cation exchange occurs, the exposure layers of MnS are replaced by Cu_2−δ_S which make the MnS be insulated from the solution. According to the E-pH diagram^7^,^22^, the equilibrium potential of Cu_2−δ_S is much higher than that of MnS in a neutral environment and Cu_2−δ_S can stably preserve in a wide range near *E*_ORR_, Therefore, the nano-galvanic cells was effectively invalided and the pitting potential of the alloy was enhanced by such a chemical decoration.

Ever since the MnS inclusion was identified as the initiation site for pitting corrosion, several methods have been tried in engineering to remove surface MnS inclusions, such as pickling, laser and high current beam modification. These approaches are useful but have considerable deficiencies. In contrast, the chemical modification via cation exchange reaction in the present study exhibits a much more feasible methodology to improve pitting corrosion resistance of stainless steels, since it is easy to operate for variant dimensions of components. Hence, this study provides a low-cost approach via an atomic scale decoration to improve the corrosion resistance of steels in a volume-treated manner, and has a great signification in improving the integrated properties of stainless steels serving in wet environments.

## Methods

### Bulk sample preparation

Commercial 316F type stainless steel with high-sulphur content (0.16%) was chosen because it can provide a large number of MnS inclusions for analysis. The steel was made by Nippon Steel and Sumikin Stainless Steel Corporation. 316L type stainless steel was made by Baosteel Corporation in Shanghai, China.

The hot-rolled steel rod was cut into sheets with the same size along the rolling direction. The samples were carefully polished with standard SiC grinding papers. The samples were then lightly polished using 1 μm diamond paste until there was no visible scratch on the surfaces. After exposure in air for 12 hours, the samples were immersed in a Cu-containing solution at room temperature for different periods. Those treated samples were cleaned by distilled water and dried for the subsequent electrochemical tests and other structural characterization (SEM, EPMA).

### TEM sample preparation

The samples cut from the steel rod were ground using variant grit silicon carbide papers, polished with diamond paste, and finally thinned by ion-milling. After the first-round TEM observation, some of the specimens were plasma-cleaned and then immersed into 1 mol/L NaCl solution for immersion-corrosion test. The TEM specimens after immersion were quickly cleaned in distilled water and methanol, dried, and transferred into the TEM for further investigation.

The plan-view TEM samples for analyzing the structural and chemical information of Cu_2−δ_S were prepared by ion-milling and treated in 0.05 mol/L CuSO_4_ solution at room temperature after first-round TEM observation.

The cross-sectional sample, used for determining the thickness of reaction layer, was prepared by the conventional methods. Two pieces of 316F stainless steel samples with polished surfaces were immersed in 0.05 mol/L CuSO_4 _solution for 1 hour. The treated surfaces of two samples were bonded face-to-face and then thinned by grinding, dimpling and ion-milling.

### TEM, SEM and EPMA characterizations

A Tecnai G^2^ F30 transmission electron microscope, equipped with HAADF detector and X-ray dispersive spectrometer systems, was used at 300 kV for diffraction, HAADF imaging, composition analysis, and 3D tomography. Microstructure characterizations of Cu_2−δ_S were performed using a Titan G^2^ 60-300 TEM equipped with double spherical aberration (Cs) correctors. A SUPRA35 field emission scanning electron microscope was used to investigate the morphology of MnS inclusions. The surface composition of the bulk materials was analyzed using an electron probe X-ray microanalyzer (EPMA: Shimazu Seisakusho EPMA-1610).

### Electrochemical test procedures

A traditional three-electrode system was used in electrochemical experiment. The working electrode is stainless steel specimen, Pt counter electrode and SCE (saturated with KCl) reference electrode. AUTOLAB PGSTAT302N electrochemical workstation was used in potentiodynamic polarization curve measurements with a scan rate 1 mV/s. All the tests were stopped when the anodic current density reached the value of 1 mA/cm^2^. The tested solution of 0.5 mol/L NaCl electrolytes was maintained at 28°C with electric-heated thermostatic water bath.

The potentiodynamic polarization curve includes the cathodic and anodic branches. In the anodic branch, there exists a well-defined inflexion, above which anodic current density sharply increase with potential, meaning stable pitting corrosion occurs. The potential value corresponding to that inflexion is identified as pitting potential (*E*_pit_).

## Author Contributions

This project was conceived by X.L.M.; TEM experiments were performed by Y.T.Z., B.Z. and S.J.Z.; electrochemical experiments were carried out by B.Z., Y.T.Z., J.W. and X.Y.S.; all the authors participated in discussion, interpretation of the data, and producing the final version of this paper.
